# Current Knowledge on Mechanisms Preventing Photosynthesis Redox Imbalance in Plants

**DOI:** 10.3390/antiox10111789

**Published:** 2021-11-09

**Authors:** María-Cruz González, Francisco Javier Cejudo, Mariam Sahrawy, Antonio Jesús Serrato

**Affiliations:** 1 Instituto de Bioquímica Vegetal y Fotosíntesis, Universidad de Sevilla-Consejo Superior de Investigaciones Científicas (CSIC), Avda. Américo Vespucio 49, 41092 Sevilla, Spain; fjcejudo@us.es; 2 Departamento de Bioquímica, Biología Celular y Molecular de Plantas, Estación Experimental del Zaidín, Consejo Superior de Investigaciones Científicas (CSIC), 18008 Granada, Spain; mariam.sahrawy@eez.csic.es

**Keywords:** thioredoxins (TRX), photosynthesis, redox, NADPH thioredoxin reductase C (NTRC), non-photochemical quenching (NPQ), cyclic electron flow (CEF), ferredoxin/PGR5/PGRL1-dependent plastoquinone reductase (PGR5/PGRL1), NADH dehydrogenase-like complex (NDH)

## Abstract

Photosynthesis includes a set of redox reactions that are the source of reducing power and energy for the assimilation of inorganic carbon, nitrogen and sulphur, thus generating organic compounds, and oxygen, which supports life on Earth. As sessile organisms, plants have to face continuous changes in environmental conditions and need to adjust the photosynthetic electron transport to prevent the accumulation of damaging oxygen by-products. The balance between photosynthetic cyclic and linear electron flows allows for the maintenance of a proper NADPH/ATP ratio that is adapted to the plant’s needs. In addition, different mechanisms to dissipate excess energy operate in plants to protect and optimise photosynthesis under adverse conditions. Recent reports show an important role of redox-based dithiol–disulphide interchanges, mediated both by classical and atypical chloroplast thioredoxins (TRXs), in the control of these photoprotective mechanisms. Moreover, membrane-anchored TRX-like proteins, such as HCF164, which transfer electrons from stromal TRXs to the thylakoid lumen, play a key role in the regulation of lumenal targets depending on the stromal redox poise. Interestingly, not all photoprotective players were reported to be under the control of TRXs. In this review, we discuss recent findings regarding the mechanisms that allow an appropriate electron flux to avoid the detrimental consequences of photosynthesis redox imbalances.

## 1. Introduction

Most of life on Earth is sustained by photochemical reactions. In general terms, in the so-called linear electron flow (LEF), photosynthetic light reactions involve three multi-protein complexes: photosystems (PS) II and I, and the cytochrome *b*_6_*f* (Cyt *b*_6_*f*) complex [1,2]. PS are associated with light-harvesting complexes (LHCs), which are responsible for sunlight absorption in plants and green algae [3,4]. LEF starts with the photo-induced water oxidation that donates electrons to PSII and ends with the ferredoxin (Fd) reduction by PSI. Additionally, a balancing cyclic electron flow (CEF) works together with LEF to fine-tune the whole photosynthetic process [5,6]. CEF depends on additional photosynthetic complexes: the NADH dehydrogenase-like (NDH) complex and/or the ferredoxin/PROTON GRADIENT REGULATION 5 (PGR5)/PGR5-LIKE PHOTOSYNTHETIC PHENOTYPE 1 (PGRL1) complex.

Oxygenic photosynthesis provoked the rise of molecular oxygen (O_2_) in the atmosphere approximately 2.3–2.4 billion years ago [7,8]. Exposition to higher levels of O_2_, and its derivative reactive oxygen species (ROS), led to a massive extinction event [9]. From then on, living organisms have taken advantage of ROS to regulate their own development [10,11]. In plants, both enzymatic and non-enzymatic systems scavenge excess ROS thus preventing ROS-derived damage to different cell components, such as DNA, lipids and proteins [12]. In chloroplasts, ROS play a key role in the photoinhibition of PSII under high light, hindering its repair through the inhibition of D1 translation [13]. However, despite their potentially harmful effects, ROS derived from photosynthesis [14,15] have a relevant role as signalling molecules for the regulation of chloroplast processes with an important impact on plant development and acclimation to environmental stress [11,14,15,16,17].

To preserve plant performance under adverse environmental conditions, the photosynthetic electron transport chain (PETC) must be finely tuned according to internal and external signals. Plant photosynthesis allows the use of reducing equivalents, generated by light-driven extraction of electrons from water, to support biosynthetic pathways, including CO_2_ fixation or nitrogen assimilation. In general terms, PETC performance greatly depends on a proper balance between absorbed light and the metabolic demand for chemical energy. Rapid environmental changes can overflow PETC causing the accumulation of ROS, which may damage the most sensitive molecular components of the photosynthetic machinery [13]. When redox imbalance occurs in chloroplasts, for instance, due to strong fluctuations in light intensity, protective mechanisms are triggered to preserve redox homeostasis. As previously mentioned, many of these mechanisms rely on scavenging over-accumulated ROS [12]. At the same time, an additional strategy consists in rebalancing photosynthesis to cope with unfavourable growth conditions, thus preventing an oxidative burst and its harmful outcomes through several conserved regulatory mechanisms, which include (i) balancing the NADPH/ATP ratio with the cyclic electron flow (CEF) carried out by the chloroplast NADH dehydrogenase-like (NDH) and the Fd/PGR5/PGRL1-dependent PQ reductase (PGR5/PGRL1) complexes, (ii) dissipating excess energy by non-photochemical quenching (NPQ), (iii) the appearance of new quenching sites in PSII peripheral antenna, (iv) redistribution of excitation energy through state transitions dependent on the phosphorylation of LHCII, (v) specific removal and repair of PSII damaged protein components, such as D1 and the extrinsic protein PsbO, and (vi) regulating the plastoquinone reduction by the plastid terminal oxidase (PTOX).

Photosynthetic complexes located at the chloroplast thylakoid membrane define two fine-tuned interconnected sub-compartments. While intrinsic proteins face both the thylakoidal lumen and the stroma, extrinsic proteins face either one or another side and, consequently, these photosynthesis complexes interact in synchrony within two physically separated protein networks. Regarding redox poise and pH, the stroma is a more reducing and basic environment than the thylakoid lumen [18]. Because sunlight intensity is continuously changing in nature, photosynthesis control (and protection) largely relies on fast regulatory mechanisms involving redox-based post-translational modifications (PTMs), which take place at both sides of thylakoid membranes in a coordinated manner [19]. When proteins are exposed to ROS, sulfhydryl groups (-SH) of Cys residues can be oxidised to sulfenic acid (-SOH) [20]. The occurrence of another Cys near this group can lead to the formation of a covalent intra- or intermolecular disulphide bond [21]. Usually, this PTM has important effects on protein conformation, activity and even stability, hence becoming a dynamic regulatory mechanism in some proteins that can switch between two redox states (thiol/disulphide) or form multiprotein complexes [22,23,24]. Some members of the chloroplast thioredoxin (TRX) family were reported to be key players in the redox regulation of light-dependent processes [25,26,27,28]. TRXs are redox signalling proteins with an active site containing two Cys separated by two amino acid residues (CXXC). These redox proteins are prone to transfer electrons to target proteins and their enzymatic mechanism implies a dithiol/disulphide interchange [29]. In chloroplasts, these proteins are reduced by the Fd-TRX reductase (FTR), which receives electrons from photosynthetically reduced Fds [30]. Classic TRXs, with the conserved active site WCG/PPC, form a multigenic family composed of different types (*f*, *m*, *x*, *y* and *z*) [31,32]. In *Arabidopsis thaliana*, the *f*- and *m*-types are the most abundant TRX isoforms in the chloroplast [33] and participate in the regulation of important processes such as the Calvin–Benson cycle and the photosynthesis light reactions [34,35,36]. Chloroplast non-classic TRXs include a particular protein named NADPH TRX reductase C (NTRC), which receives reducing equivalents from NADPH [37,38] and was proposed to be a key player in chloroplast redox homeostasis [39,40]. Interestingly, classic TRXs and NTRC are restricted to the chloroplast stroma, indicating that redox regulation is particularly relevant in this chloroplast compartment in line with recent reviews that have highlighted the importance of redox regulation of stromal processes [34,35,41]. Besides stromal TRXs, lumenal TRX-like counterparts, such as HCF164, anchored to the thylakoid membrane, were identified [42]. HCF164 can receive reducing equivalents from stromal TRXs *m* through the thylakoid-membrane protein CCDA [19,43,44]. Therefore, according to the current model, the lumenal system CCDA/HCF164 depends on the stromal FTR/TRX redox system. Nevertheless, the interrelation between stromal and lumenal TRXs in the redox regulation of light reactions of photosynthesis is largely unknown.

As photosynthesis can be considered the most important biological process for life on Earth, the knowledge of the signalling networks, including redox regulation, that operates in the control of light reactions of photosynthesis, is key to understanding the life-adaptation success on our planet. Thus, the aim of this review is to unveil an integrative view of the different regulatory mechanisms rebalancing photosynthesis light reactions under unfavourable conditions. In this regard, TRXs are key regulatory players receiving information about the redox state of the PETC, conveying back this information to orchestrate the whole photosynthetic process. However, other mechanisms, with so far no reported TRX-dependent redox regulation, which participate in the balance of redox homeostasis in green tissues, will also be discussed.

## 2. Balancing Photosynthesis through Cyclic Electron Flow

CEF has evolved to divert electrons back to PETC to enhance H^+^ pumping to boost the proton motive force (PMF) across the thylakoid membranes [45]. PMF has two components, the proton gradient (∆pH) and the membrane potential (∆Ψ), and is responsible for the ATP synthesis by the ATP synthase of the thylakoid membrane, a process that is subjected to redox regulation [46,47,48]. Other thylakoid-localised transport processes, such as the K^+^ exchange antiporter 3 (KEA3), operate to harmonise PMF with metabolic requirements. KEA3 acts modulating ATP synthesis hence relaxing ∆pH by proton export from the thylakoid lumen [49]. KEA3 activity might be controlled via the stroma-located C-terminal domain, possibly by monitoring the NADPH/NADP^+^ ratio [50]. Wang and co-workers have also proposed a second type of redox regulation involving an N-terminal Cys residue facing the lumen side [50].

**Figure 1:**
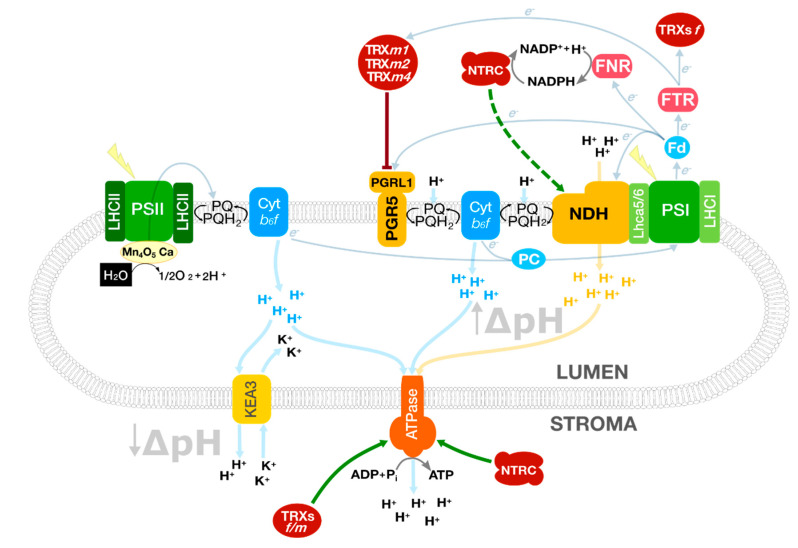
Photosynthetic processes involved in balancing the NADPH/ATP ratio in *Arabidopsis* chloroplasts. TRXs receive redox equivalents from the linear electron flow and control the cyclic electron flow to fine-tune the proton motive force and the ATP synthesis. At the same time, KEA3 modulates the ATPase activity controlled by TRXs *f/m* and NTRC. The soluble electron carriers ferredoxin (Fd) and plastocyanin (PC) operate at both sides of the thylakoid membrane connecting PSI with the CEF complexes and Cyt *b*_6_*f*, respectively. Green arrows represent direct activation mediated by TRXs or NTRC; truncated red lines, inhibition. A dashed line indicates a hypothetical interaction. Protein complexes were schematised for a better understanding.

The question arising is why plants have two CEF systems, NDH and PGR5/PGRL1 complexes, both of them having a role in protection against light stress. In chloroplasts, multiple interconnected biosynthetic pathways are operating at the same time. In general, these processes are NADPH and ATP consuming, but the NADPH/ATP ratio to support each metabolic pathway is diverse and this ratio must be adapted to specific needs (i.e., Calvin–Benson cycle, nitrogen and sulphur assimilation, lipid biosynthesis, isoprenoid precursor biosynthesis, etc.). Reaching a proper balance between NADPH and ATP, integrating light (energy input), plant development and cell catabolism might involve dynamic processes acting at the photosynthesis level (Figure 1). According to this reasoning, it would be interesting to investigate and shed more light on the role of CEF in plant physiology/metabolism under non-stress conditions.

### 2.1. The NDH Complex

The *Arabidopsis* NDH complex is composed of 29 subunits (11 of which are plastid-encoded) grouped in five subcomplexes [51]. The chloroplast NDH complex, which has a molecular mass of approximately 700 kDa, shows homology with the respiratory complex I of bacteria and mitochondria. This complex is present as a monomeric complex associated with PSI [52]. Some authors have recently determined that in A. thaliana plants grown under non-stressing conditions the PSI:NDH ratio is about 45.4 [53]. Concerning redox regulation of NDH activity, it was proposed that NTRC exerts an activating effect, [54] whereas TRX *m*4 was suggested to be a negative regulator [55] (Figure 1). Whereas several NDH subunits were identified in a co-immunoprecipitation assay using anti-NTRC antibodies, supporting their redox regulation [54], the mechanism of TRX *m*4-dependent NDH regulation remains to be determined [55]. Since, as far as we know, no thiol/disulphide mechanisms were proposed to directly regulate NDH activity, the post-translational redox regulation of NDH remains elusive. Nevertheless, though indirectly, redox signalling affects NDH regulatory proteins acting at transcriptional or translational levels. For instance, it is known that hydrogen peroxide can trigger the activity of the NDH complex in barley [56] and that low levels of ascorbic acid or reduced glutathione downregulate genes coding for NDH subunits [57]. Paradoxically, though many years ago phosphorylation was described to activate the NDH complex [58], no direct redox regulation has been proved so far for this photosynthetic complex. The question is whether NDH- and PGR5/PGRL1-dependent CEF integrate different types of chloroplast cues to balance the NADPH/ATP ratio under a broad range of environmental and developmental situations. Interestingly, several orchid species, plants that have a heterotrophic phase in their life cycle, have lost the NDH complex, which led to propose that the loss of NDH complex might be necessary for these plants [59]. The question is whether this apparently TRX-independent process is related to the role of NDH in non-photosynthetic organs as fruits [60].

### 2.2. The PGR5/PGRL1 Complex

The PGR5/PGRL1 complex is formed by two subunits and exerts a photo-protective role against high-light mediated stress (Figure 1) [61,62]. Unlike NDH, there are solid experimental data showing the redox-regulation of PGR5 [55,63,64]. In line with this notion, it was reported that TRXs *m* collectively down-regulate the PGR5/PGRL1 complex [63]. In planta, TRX *m*4 downregulates PGR5 activity by reducing PGRL1 (Figure 1) [55]. In addition, the relevance of the regulation of PGR5/PGRL1 on the chloroplast redox state was evidenced by the recovery of enzyme reduction in the *ntrc pgr5 Arabidopsis* double mutant [65].

The cyanobacteria *Synechocystis* sp. contains PGR5 but not PGRL1 [66]. Nevertheless, in spite of the low similarity between the cyanobacterial protein SII1217 and PGRL1, both proteins might be functionally related, suggesting a putative prokaryotic origin of PGRL1. Interestingly, *Arabidopsis* PGRL1 has six cysteine residues whereas cyanobacterial SII1217, which has not been shown to be redox-regulated, has only three [63,64]. Anyway, *Synechocystis* mediates the non-photochemical reduction of PQ possibly via CEF through the NDH-1 complex [67,68]. Another aquatic organism, the marine angiosperm *Zostera marina*, conserves the two CEF systems and shares with land plants a similar response to respond to excess radiation [69].

### 2.3. Ferredoxins: Active Players Balancing Linear and Cyclic Electron Flows?

Chloroplast Fds are small proteins containing a [2Fe:2S] cluster with low redox potentials. These proteins regulate electron partitioning in plant chloroplasts by transferring electrons from photo-reduced PSI to different stromal proteins such as Fd NADP^+^ reductase (FNR) or FTR, as well as to the thylakoid-located CEF systems NDH and PGR5/PGRL1 [70]. *Arabidopsis* harbours four chloroplastic Fd isoforms, namely FD1 (AT1G10960), FD2 (AT1G60950), FDC1 (AT4G14890) and FDC2 (AT1G32550) [71,72]. FD1 and FD2 account for 7% and 90% of the total leaf Fd, respectively [73]. The presence of several Fd isoforms in plants suggests the existence of specific targets for these enzymes. In this regard, it was hypothesised that FD1 would contribute to CEF and FD2 to LEF [74,75,76,77]. FDC1 and FDC2 have an additional extension at the C-terminus, near their active sites, as well as higher redox potentials than FD2, hence FDC1 and FDC2 can be considered atypical Fds [71,72]. Unlike FD1 and FD2, FDC1 is not able to interact with FNR; nevertheless, it can interact with the CEF complexes NDH and PGR5/PGRL1 or with FTR [72]. These results suggest a role of FDC1 in partitioning providing electrons to specific chloroplast processes. Although there is no experimental evidence so far, the possibility cannot be discarded that FDC2 might have a similar function to FDC1. Its functional significance in plants was proven in rice, where a mutation in the FDC2 ortholog HDY1 provokes leaf yellowing and a delay in flowering time [78].

In photosynthetic organisms, the docking site at PSI, formed by the subunits PsaD and PsaE, allows the electronic transfer between Fds and PsaC [79]. Remarkably, *Arabidopsis* has two isoforms of PsaD and PsaE [72], thus, it is tempting to speculate that the combination of these isoforms might constitute auxiliary docking sites for chloroplast Fds. If this were the case, the donor site of PSI would also play an active role in photosynthesis electron partitioning and redox regulation.

## 3. Redox Regulation of Non-Photochemical Quenching

**Figure 2:**
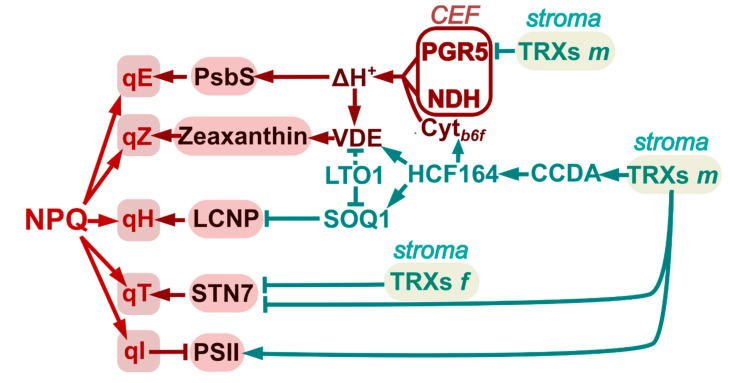
Schematic representation of the redox regulation mediated by TRXs *f* and *m* of the NPQ components in *Arabidopsis*. The type of interaction is either represented in green (redox type) or in red (non-redox type). Arrows represent activation; truncated lines, inhibition. A dashed line indicates a hypothetical interaction.

Light energy reaching the chloroplast can be either emitted as chlorophyll fluorescence or quenched by photochemical (qP) and non-photochemical mechanisms (qN or NPQ). When qP is not sufficient to assimilate all the absorbed energy, a fraction of it must be released as heat by NPQ. NPQ has different components: qE (energy-dependent quenching), qZ (zeaxanthin-dependent quenching), qT (state-transition quenching), qI (photo-inhibitory quenching) and qH (sustained and slowly reversible quenching) [80,81,82]. Behind all these photoprotective mechanisms, there is a dynamic redox network in which TRXs play active roles [28]. It follows now a discussion of the relevance of TRXs in the regulation of the different NPQ components (Figure 2).

Defects in NDH can affect ∆pH formation and lead to impaired activation of NPQ energy-dependent quenching [83]. The pigment zeaxanthin is another key component of NPQ in plants, being responsible for pH-independent qZ, and energy-dependent quenching (qE). In excess light, the high pH gradient favours protonation of PsbS subunit of PSII, triggering qE and activating the enzyme violaxanthin de-epoxidase (VDE), which catalyses the conversion of violaxanthin to zeaxanthin in the thylakoid lumen. This enzyme is stimulated by the thylakoid lumen acidification upon illumination and is active in its oxidised state [84,85]. As TRXs *m* deliver electrons into the thylakoid lumen through CCDA and HCF164 [19,44], these TRXs can indirectly regulate enzymes such as VDE (Figure 2). VDE was identified as a putative target of a lumenal disulphide-forming enzyme termed Lumen Thiol Oxidoreductase1 (LTO1), suggesting that it could play a key role in the redox-dependent regulation of zeaxanthin levels [86]. The other enzyme participating in the xanthophyll cycle, zeaxanthin epoxidase (ZE), which catalyses the conversion of zeaxanthin and antheraxanthin to regenerate violaxanthin, is also regulated by TRXs. Mutant plants lacking TRXs *m* accumulate higher levels of aggregated/inactivated ZE and zeaxanthin [87]. In addition, NTRC deficient plants showed increased zeaxanthin levels and elevated qE; however, though NTRC can reduce ZE aggregates in vitro, no alteration of ZE redox state was observed in *ntrc* mutant plants. Rather, the increased ∆pH under low and moderate light intensities in *ntrc* plants seems to be responsible for the activation of VDE [88].

### 3.1. Photo-Protective Quenching in LHCII: Is Lipocalin Subjected to Redox Regulation?

**Figure 3:**
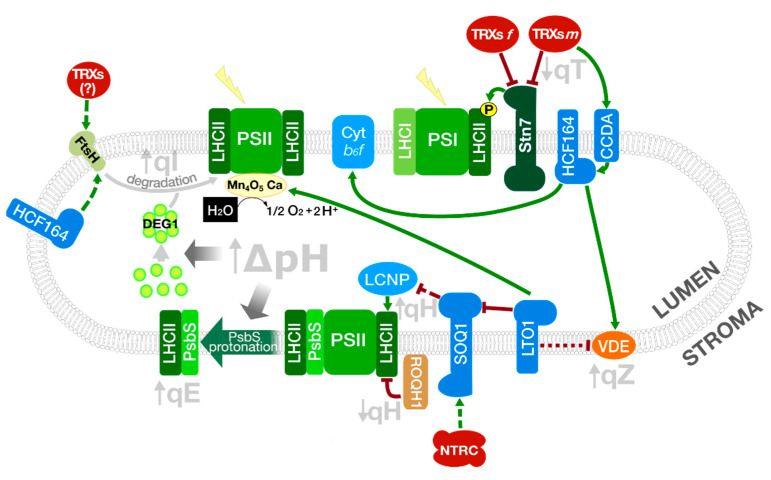
Regulation of the NPQ components in *Arabidopsis* chloroplasts. TRXs *m* are key redox players in the regulation of NPQ in plants as they transfer reducing equivalents into the thylakoid lumen through the proteins CCDA and HFC164. The thylakoid protein LTO1, with an oxidant role, contributes to maintaining the redox homeostasis in the chloroplast lumen. Green arrows represent activation mediated by TRXs or NTRC; truncated red lines, inhibition. A dashed line indicates a hypothetical interaction. Protein complexes were schematised for a better understanding.

A new component of pH-independent NPQ, sustained quenching or qH, that precedes PSII damage and repair, was recently identified in a search for suppressors of *npq4* mutant plants lacking PsbS [82,89] (Figure 3). This ∆pH-independent mechanism, similar to that in evergreens, is dependent on a plastid lipocalin (LCNP), localised in the lumen, and related to the appearance of new quenching sites in LHCII. LCNP is negatively regulated by SOQ1 (Suppressor of quenching 1), a TRX-like/β-propeller protein, through a mechanism that is so far unknown [82]. Since Cys residues of the TRX-like lumenal active-site motif of SOQ1 are required for the suppression of qH, and LCNP contains six conserved Cys, a putative redox-dependent regulation of LCNP by SOQ1 was suggested. However, the down-regulation of SOQ1 under drought stress and the inability to reverse the electrophoretic mobility of LCNP with DTT in *soq1* mutants, argues against it, suggesting rather an increased activity of LCNP due to the decrease in SOQ1 levels under stress conditions [82]. Nevertheless, the finding of SOQ1 as a possible NTRC interactor [54] and the recent identification of LTO1, in a genetic screen for suppressors of *soq1 npq4* by Bru and co-workers (2020), has raised again the question of a possible redox regulation of LCNP [90]. The participation of NTRC in the down-regulation of qH has recently been proposed [91].

Recently, a new player in qH regulation present in all plastid-containing organisms, ROQH1 (RELAXATION OF QH1), with an antagonistic function to LCNP, was identified. ROQH1 is a stroma lamella membrane-associated protein, belonging to a NAD(P)H-dependent atypical short-chain dehydrogenase/reductase (SDR) subfamily, that produces a dose-dependent relaxation of qH, turning the LCNP created quenching sites back into light-harvesting sites [92].

While SOQ1 is present in *Chlamydomonas* and *Synechocystis* sp. as two independent proteins corresponding to the HAD and the NHL/TRX-like domain of SOQ1, and ROQH1 homologues were identified in *Synechocystis* sp. PCC 6803, the low sequence conservation between LCNP homologues makes it difficult to analyse whether qH is a conserved mechanism from cyanobacteria.

### 3.2. Redistribution of Excitation Energy between the PSs: Role of Redox Regulation of LHCII Kinase and Cyt b_6_f Assembly in State Transitions

Light quality in the natural environment is variable, thus chloroplasts require a dynamic system allowing the distribution of excitation energy between the two photosystems, preventing imbalance in PETC in photosynthetic organisms, and avoiding photoinhibition especially under fluctuating light conditions. In plants and algae, the re-distribution of excitation energy is dependent on state transitions mediated by the phosphorylation of the light-harvesting complex II (LHCII) by a serine/threonine LHCII kinase, known as Stt7 or Stn7 in Chamydomonas reinhardtii and A. thaliana, respectively. The regulation of LHCII phosphorylation and its effect on the migration of LHCII from PSII to the PSI is known for many years: LHCII kinase is activated by reduced PQ in low light and inactivated by TRX in high light [93]. More recently, a relevant role for the Rieske iron-sulphur protein of the Cyt *b_6_f* complex was proposed, in which the movement of the protein within this complex, after binding of reduced PQ, generates a conformational change in the complex that in turns activates LHCII kinase [94]. The physical interaction of Stt7 with the Cyt *b_6_f* complex and PSI was demonstrated by co-immunoprecipitation experiments in Chlamydomonas and the Rieske protein was identified as the interactor with Stt7 [95].

The topology of Stt7 was analysed using a tagged protein, revealing that the protein contains a transmembrane domain, with the kinase activity at the stromal side and the N-terminal region, containing the two conserved Cys within algae and plant LCHII kinases, in the lumen. The disulfide bond between these Cys is essential for the phosphorylation of LHCII, suggesting that redox regulation might be critical for Stt7 activity [95]. This regulation could be mediated by luminal TRX-like proteins, such as HCF164 and CCDA, which were proposed to participate in the transduction of TRX signals from the stroma [42,96]. The work of Shapiguzov et al. (2016) revealed, however, that the disulfide bridge in both Sst7 and Stn7 is maintained during activation and inactivation of the kinases, which questions the redox regulation of LHCII kinase [97]. It was suggested that two conserved Cys residues of LHCII kinase located in the stroma, but not conserved in algae, could be regulated by the FTR/TRX system, however, the analysis of the specificity of TRXs *f* and *m* in the process has given contradictory results. While in vitro analysis showed direct interaction between TRX *f* and Stn7 [98] and an inhibitory effect of both TRX *f* and *m* on LHCII phosphorylation was shown [99], the analysis of *trxm1m2* mutants under fluctuating light conditions not only suggested the activation of Stn7 as a compensatory mechanism to increase photosynthesis during low light periods, but also the essentiality of this regulation for complete activation of photosynthesis during high light periods [100]. More recently, studies performed with tobacco plants have revealed that plants over-expressing TRX *m*, but not TRX *f*, showed a loss of LHCII phosphorylation under low light, suggesting a role for TRX *m* in the deactivation of Stn7. Moreover, since the phenotype of TRX *m* over-expressing plants mimics that of wild-type plants under high light, when LHCII is not phosphorylated, the results suggest a role for TRX *m* in the deactivation of Stn7 under high light [101]. Recently, it was reported that the altered chloroplast thiol redox state in *ntrc* mutants and NTRC over-expressing plants provokes a re-distribution of excitation energy between the two PSs, altering state transitions, through a mechanism that is probably independent of Stn7 but rather mediated by CP29.3, a monomeric LHC protein with a conserved Cys residue [91]. The precise role of redox regulation on the deactivation of LHCII kinase and the conservation of this mechanism of regulation in both planta and algae remains to be determined.

It is worth mentioning that the importance of Stn7 in state transitions in flowering plants is not clear since loss-of-function of this protein does not result in significant alterations of plant development. Instead, only when the *stn7* mutation is combined with mutations leading to a decreased pool of PQ the growth rate and state transitions are affected, showing that these transitions are critical when linear electron flow is altered [102,103]. The change in the redox status of the PQ pool provokes long-term changes in gene expression of *Lhcb1*, probably adjusting the antenna size as an additional mechanism to balance the use of excitation energy between the two PSs [104]. The regulation of Stn7 by the PQ redox state [102] supports this hypothesis.

Remarkably, the participation of HCF164 in Cyt *b*_6_*f* assembly was also proposed [42]. Apo-cyt *f* and the haem groups are both synthesised at the stromal side of the thylakoid membrane and are transported independently to the lumen. Once in the lumen, the reduced haem group is attached to the binding site of Cyt *f* by means of a thioether bond. For this, the apo-cyt must be maintained in a reduced state. The transfer of electrons from the stroma to the lumen and the preservation of apo-cyt *f* in a reduced state is carried out via CCDA and HCF164, as suggested by the analysis of *Arabidopsis ccdA* and *hcf164* mutants, which show defective Cyt *b*_6_*f* accumulation [42,43]. In *C. reinhardtii* CCS5, and probably CCS4, homologues of HCF164, could reduce the haem binding site in apo-cyt *f* [96]. Recent studies pointed out the role of TRXs *m* in the transfer of electrons, needed for Cyt *f* reduction, from the stroma to the lumen through HCF164 [19,96].

Finally, the formation of the iron-sulphur cluster in the Rieske protein, PSI and Fd, depends on the activity of two class-two GRXs, that are unable to reduce disulphide bridges. In chloroplasts, similarly to bacteria, dimers of GRX14 and GRX16, together with the scaffold proteins BOL1 and BOL4, can bind 2Fe:2S clusters and consequently transfer them to Fd in vitro [105,106].

### 3.3. Photodamage and Repair of PSII: Redox Regulation of D1 Degradation and PsbO Stability

PSII is highly susceptible to excess light, which leads to its irreversible damage, provoking degradation of its core proteins, such as D1. D1 is rapidly replaced in the PSII repair cycle so that photoinhibition only occurs when the rate of repair is slower than the rate of damage [107]. In contrast, the recovery of photo-inhibited PSI occurs very slowly. For this reason, PSI is protected from photoinhibition by several mechanisms, which include a decreased rate of electron transfer to PSI due to PSII degradation and down-regulation of electron transport through Cyt *b*_6_*f*, probably mediated by PGR5/PGRL1 [62]. The relevance of the latter in the protection of PSI from photoinhibition is revealed by the results by Lima-Melo and co-workers (2018), showing that *pgr5* mutant plants contained decreased levels of PsaB core subunit and severely decreased Fd reduction under high light conditions. The recovery of PSI in these plants seems to be dependent on the reorganisation of the light-harvesting antenna, through increased phosphorylation of LHCII. In addition, a “reserve” PSI* complex, lacking LHCI antennae and peripheral subunits, could help to support PETC under PSI photoinhibition [108].

PSII consists of more than 30 integral membrane proteins, including the catalytic reaction centre and the chlorophyll-binding proteins, stabilised by extrinsic proteins located at the lumenal side that form part of the Oxygen Evolving Complex (OEC). This complex is formed by four extrinsic proteins, PsbO, PsbP, PsbQ and PsbR. PsbO interacts with several core subunits of PSII and seems to provide a basal structure to which the other OEC subunits are bound [109].

The damage of proteins in the reaction centres of the PSII and their repair constitute the photo-inhibitory quenching, qI, component of NPQ (Figure 2 and Figure 3). Damaged D1, and in some cases D2, CP43 and PsbH of the PSII core, are subjected to Deg-dependent proteolysis under conditions of PSII photo-damage, by Deg7, which is associated with the stromal side of the thylakoid membranes. In addition, a role for stromal and lumenal Deg proteases in the degradation of PSII core proteins was suggested [107,110]. Since damaged D1 protein is associated with lower photosynthetic activity, a mechanism for the degradation of the damaged protein is essential to maintain photosynthetic performance. However, the damage to the Mn-cluster seems to be a primary step leading to D1 degradation mediated by the activity of these proteases [111,112]. As a first step for the repair of PSII and the degradation of specific core proteins, the PSII dimers or supercomplexes, located to the grana regions of the thylakoid, must disaggregate to monomers and migrate to stromal thylakoids [113]. Monomerisation depends on the phosphorylation of the above-mentioned PSII core proteins by Stn8 and Stn7 kinases, which leads to grana de-stacking and easier access to the repair machinery [107]. The degradation of D1 requires, in addition, the proton gradient-dependent formation of Deg1 homo-hexamers [113] and FtsH metalloprotease oligomerisation (Figure 3).

FtsH is a membrane-anchored ATP-dependent zinc metalloprotease, also involved in the biogenesis and repair of the PSII, that cooperates with Deg proteases in the degradation of D1. Two types of FtsH subunits, termed A (FtsH1 and FtsH5) and B (FtsH2 and FtsH8), possibly form a transient hetero-hexameric complex in the lumen of plant thylakoids. All of these subunits have an N-terminal transmembrane domain, a C-terminal extension to the stroma, with ATPase activity, that pulls integral proteins out of the membrane, and a protease domain [114]. FtsH2 and FtsH8 are required for the formation of an active complex, as suggested by the albino phenotype of *ftsh2 ftsh8* mutant plants [115].

Under photo-inhibitory conditions, FtsH cooperates with Deg proteases in the degradation of D1 in the PSII repair cycle. It was suggested that Deg mediated proteolysis of D1 might create recognition sites for FtsH, which degrades D1 in a processive manner, a process initiated at the stroma exposed N-terminus side of the protein [116,117]. In C. reinhardtii, it has recently been shown that light increases FtsH activity through redox control. The FtsH oligomers, formed by type A FtsH1 and type B FtsH2, disappear when membrane protein extracts were treated with a reductant, increasing their proteolytic activity. Newly formed FtsH oligomers after incubation with oxidising reagents inhibit FtsH dependent proteolysis. The reduction of FtsH oligomers in high light suggests that this could be part of an initial response to photo-inhibitory conditions leading to PSII degradation. Since all conserved Cys in FtsH are on the stromal side, the participation of stromal TRXs in the in vivo reduction of FtsH was suggested [50]. The identification of FtsH8 and FtsH2 as HCF164 targets in the lumen raised the possibility that these proteases are also subjected to redox regulation mediated by HCF164 [44]. However, this possibility could be ruled out since most Cys in FtsH, which might be redox-regulated, lie at the stromal ATPase domain of FtsH. Therefore, the residues involved in this regulation and the redox system responsible for it remain to be determined.

In addition to a possible role of redox control on D1 degradation in PSII repair, the role of TRXs or TRX-like proteins in the assembly of PSII was suggested by different proteomic approaches [118,119]. Interestingly, immunoblot analyses using FLAG-tagged m-type TRXs and pull-down assays have shown that TRXs *m*1, *m*2 and *m*4 can interact directly with D1, D2 and CP47 and co-migrate with PSII assembly intermediates, suggesting their implication in PSII assembly [25].

A detailed description of the process of PSII assembly and repair, including the roles played by the different stromal, thylakoid-bound and lumenal auxiliary proteins assisting PSII repair, recently reviewed by Järvi et al. (2015) [107], is outside the scope of this manuscript. However, we would like to point out the role played by, the previously mentioned, thylakoid bound LTO1 in PSII accumulation. Since *lto1* mutants showed decreased levels of PSII core proteins, a role for LTO in D1 degradation is suggested [120].

As mentioned before, damage to the Mn-cluster seems to pave the way for D1 degradation. The Mn_4_Ca cluster, associated with extrinsic loops of integral membrane subunits of the PSII, is part of the water-oxidising complex responsible for the oxidation of water to dioxygen [121]. The stability of this cluster is influenced by some extrinsic proteins, such as PsbO, present in all oxygenic photosynthetic organisms.

In the thylakoid lumen, redox regulation of PsbO, which contains two Cys residues that form a disulphide bridge, was suggested. This regulatory mechanism would modulate PsbO stability and function in the PSII, in which reduction of the protein-mediated by TRXs would result in its destabilisation and degradation by luminal proteases [122]. This hypothesis was supported by the identification of PsbO1 and PsbO2 of *Arabidopsis* as TRX targets [119,123,124]. However, the regulation of luminal proteins by the classical TRXs is not probable, due to their stromal location. Instead, other atypical TRXs, localised to the lumen, such as HCF164 and HCF153, could participate in PsbO1 redox regulation. In addition, lumenal LTO1, with a TRX-like domain towards the lumen, is able to oxidise PsbO in vitro [120]. The implication of the disulphide bridge on the function of PsbO remains unclear since mutation of the two Cys residues of the protein has no effect on OEC activity or its rebinding to PSII [125]. However, it results in the destabilisation of the otherwise highly ordered structure of the protein, affecting the packaging interaction and the whole protein stability [126].

In fact, the analysis of the TRX-reduced lumenal proteome showed that PsbO1 and PsbO2 are degraded in presence of TRX. Since Deg1, Deg5 and Deg8 are the only proteolytic activities in the chloroplast lumen and the degradation of PsbO was inhibited by TPCK, an inhibitor of trypsin-like serin proteases such as Deg/HtrA, a function of these proteolytic enzymes in PsbO1 degradation was proposed. Noteworthy, lumenal Deg proteases were also identified as TRX targets [119]. Indeed, spinach purified PsbO is stable in its oxidised form but, upon reduction by TRX, is cleaved by *Synechocystis 6*803 recombinant HhoA, a homologue of lumenal Deg1 protease [124]. In a recent approach, PsbO1 was shown to co-precipitate in *Arabidopsis* with Deg8 [127]. Notably, the association of PsbO to PSII protects the protein from degradation by Deg proteases [124]. Although the effect of PsbO reduction in the association of the protein to the PSII is still a matter of debate [126], the results suggest that TRX-mediated reduction of PsbO makes it more accessible to degradation [122]. Whether the reduction is related to an increase in the free form of the protein remains to be determined, as it is the redox regulation of Deg proteases.

## 4. Plastid Terminal Oxidase: A Multitasking Redox Security Valve

Plastoquinone (PQ) is not only an electron transporter in the PETC, in addition, a large fraction of this metabolite (up to 50%) is localised in other membrane systems, such as the plastoglobules and chloroplast envelopes, acting as a potent antioxidant [128]. In fact, the amount of PQ bound to the thylakoid membranes does not change under conditions that increase the levels of total PQ [129]. The antioxidant role of PQ is evident from the higher tolerance to oxidative stress of plants over-expressing SOLANESYL DIPHOSPHATE SYNTHASE 1 (SPS1), with reduced ^1^O_2_ levels and increased oxidised forms of PQ compared to wild-type plants [130].

The plastid terminal oxidase (PTOX) is a plastoquinol oxidase involved in transferring electrons from plastoquinol (PQH_2_) to O_2_ [131,132]. PTOX was proposed as an important redox sensing component in etioplasts ([133] and seems to be important in the desaturation reactions of carotenoid biosynthesis [134]. PTOX regulates the PQ pool redox state thanks to its PQH_2_ oxidase activity [135]. This protein is an important player for plant acclimation to environmental conditions. An illustrative example of its importance is the PTOX polymorphism found in some *Arabidopsis* accessions. Recent research has found that natural selection has conserved mutations providing a positive natural selection related to altitude and rainfall [136]. Interestingly, the overexpression of a chimeric rice PTOX in *Synechocystis* sp. PCC 6803 provoked a change in the PSI/PSII ratio [137]. This result suggested that PTOX might be controlling the PETC genes through the regulation of the PQ/PQH_2_ ratio. In this sense, some authors have suggested that the PQ pool might produce H_2_O_2_ as a result of the reaction of superoxide anions with PQH_2_ [138]. Similar to NDH, we wonder whether the fact that PTOX also has a physiological function in non-green plastids [133] is related to the lack of redox regulation mediated by TRXs.

## 5. Conclusions

Environmental factors, together with inherent genetic aspects, determine plant growth and development. For instance, soil features, such as nutrient availability, moisture, soil porosity or pH, may trigger long-term responses which can affect plant architecture. These changes are not reversible and highly define plant phenotype in response to more or less steady conditions. However, a second type of environmental factor, such as light, temperature or water precipitation, are far more unpredictable. In fact, light quality/intensity can be continuously changing throughout the day, challenging plants to deal with variable energy inputs for photosynthesis. At present, we are just beginning to understand the complexity of the plant signalling network behind this adaptability. Nevertheless, some decades ago, photosynthesis was rather perceived as a rigid and monotonous process as not much data about its regulation were known (with the exception of CEF). This idea has drastically changed as we currently know that multiple redox processes are concertedly acting to re-balance photosynthesis homeostasis, in which chloroplast TRXs are key redox players. Remarkably, photosynthetic organisms are equipped with complex systems able to rapidly transform the sunlight energy into redox information devoted to optimise metabolism and protecting photosynthesis in a very efficient way.

This review was focused on photosynthesis protecting mechanisms and the importance of redox PTMs for the light response. Interestingly, apart from the already mentioned TRXs or TRX-like proteins, we have to take into account that other redox players (e.g., non-classical TRXs and proteins of unknown function) are not yet on the scene. In addition, the regulatory mechanisms of some non-redox-regulated photoprotective players were discussed. The fact that these systems can operate in non-green plastids, exposed to a more oxidising molecular environment, might explain their putative independence of redox regulation mediated by TRXs. In our opinion, we are just observing the tip of the iceberg in the regulation of photosynthesis light reactions and we still have to complete this complex signalling puzzle in the coming years.

## References

[1] Govindjee D.S., Björn L.O. (2017). Evolution of the Z-scheme of photosynthesis: A perspective. Photosynth. Res..

[2] Sánchez-Baracaldo P., Cardona T. (2020). On the origin of oxygenic photosynthesis and cyanobacteria. New Phytol..

[3] Grossman A.R., Bhaya D., Apt K.E., Kehoe D.M. (1995). Light-harvesting complexes in oxygenic photosynthesis: Diversity, control, and evolution. Annu. Rev. Genet..

[4] Schmid V.H. (2008). Light-harvesting complexes of vascular plants. Cell Mol. Life Sci..

[5] Munekage Y., Hashimoto M., Miyake C., Tomizawa K., Endo T., Tasaka M., Shikanai T. (2004). Cyclic electron flow around photosystem I is essential for photosynthesis. Nature.

[6] Nawrocki W.J., Bailleul B., Picot D., Cardol P., Rappaport F., Wollman F.A., Joliot P. (2019). The mechanism of cyclic electron flow. Biochim. Biophys. Acta Bioenerg..

[7] Blank C.E., Sánchez-Baracaldo P. (2010). Timing of morphological and ecological innovations in the cyanobacteria--a key to understanding the rise in atmospheric oxygen. Geobiology.

[8] Lyons T.W., Reinhard C.T., Planavsky N.J. (2014). The rise of oxygen in Earth’s early ocean and atmosphere. Nature.

[9] Hodgskiss M.S.W., Crockford P.W., Peng Y., Wing B.A., Horner T.J. (2019). A productivity collapse to end earth’s great oxidation. Proc. Natl. Acad. Sci. USA.

[10] Schmidt R., Schippers J.H. (2015). ROS-mediated redox signalling during cell differentiation in plants. Biochim. Biophys. Acta.

[11] Mittler R. (2017). ROS are good. Trends Plant Sci..

[12] Tripathy B.C., Oelmüller R. (2012). Reactive oxygen species generation and signalling in plants. Plant Signal Behav..

[13] Nishiyama Y., Allakhverdiev S.I., Murata N. (2006). A new paradigm for the action of reactive oxygen species in the photoinhibition of photosystem II. Biochim. Biophys. Acta.

[14] Mehler A.H. (1951). Studies on reactions of illuminated chloroplasts. I. Mechanism of the reduction of oxygen and other Hill reagents. Arch. Biochem. Biophys..

[15] Asada K. (1999). The water-water cycle in chloroplasts: Scavenging of active oxygens and dissipation of excess photons. Annu. Rev. Plant Physiol. Plant Mol. Biol..

[16] Apel K., Hirt H. (2004). Reactive oxygen species: Metabolism, oxidative stress, and signal transduction. Annu. Rev. Plant Biol..

[17] Wrzaczek M., Brosché M., Kangasjärvi J. (2013). ROS signalling loops-production, perception, regulation. Curr. Opin. Plant Biol..

[18] Werdan K., Heldt H.W., Milovancev M. (1975). The role of pH in the regulation of carbon fixation in the chloroplast stroma. Studies on CO_2_ fixation in the light and dark. Biochim. Biophys. Acta.

[19] Motohashi K., Hisabori T. (2010). CcdA is a thylakoid membrane protein required for the transfer of reducing equivalents from stroma to thylakoid lumen in the higher plant chloroplast. Antioxid. Redox Signal.

[20] Miki H., Funato Y. (2012). Regulation of intracellular signalling through cysteine oxidation by reactive oxygen species. J. Biochem..

[21] Rehder D.S., Borges C.R. (2010). Cysteine sulfenic acid as an intermediate in disulfide bond formation and nonenzymatic protein folding. Biochemistry.

[22] López-Calcagno P.E., Howard T.P., Raines C.A. (2014). The CP12 protein family: A thioredoxin-mediated metabolic switch?. Front. Plant Sci..

[23] Gütle D.D., Roret T., Hecker A., Reski R., Jacquot J.P. (2017). Dithiol disulphide exchange in redox regulation of chloroplast enzymes in response to evolutionary and structural constraints. Plant Sci..

[24] Serrato A.J., Romero-Puertas M.C., Lázaro-Payo A., Sahrawy M. (2018). Regulation by *S*-nitrosylation of the Calvin-Benson cycle fructose-1,6-bisphosphatase in *Pisum sativum*. Redox Biol..

[25] Wang P., Liu J., Liu B., Feng D., Da Q., Wang P., Shu S., Su J., Zhang Y., Wang J. (2013). Evidence for a role of chloroplastic m-type thioredoxins in the biogenesis of photosystem II in *Arabidopsis*. Plant Physiol..

[26] Yoshida K., Hisabori T. (2016). Two distinct redox cascades cooperatively regulate chloroplast functions and sustain plant viability. Proc. Natl. Acad. Sci. USA.

[27] Cejudo F.J., González M.-C., Pérez Ruiz J.M. (2021). Redox regulation of chloroplast metabolism. Plant Physiol..

[28] Serrato A.J., Rojas-González J.A., Torres-Romero D., Vargas P., Mérida A., Sahrawy M. (2021). Thioredoxins *m* are major players in the multifaceted light-adaptive response in *Arabidopsis thaliana*. Plant J..

[29] Holmgren A., Björnstedt M. (1995). Thioredoxin and thioredoxin reductase. Methods Enzymol..

[30] Schürmann P., Buchanan B.B. (2008). The ferredoxin/thioredoxin system of oxygenic photosynthesis. Antioxid. Redox Signal.

[31] Serrato A.J., Fernández-Trijueque J., Barajas-López J.D., Chueca A., Sahrawy M. (2013). Plastid thioredoxins: A one-for-all redox-signalling system in plants. Front. Plant Sci..

[32] Geigenberger P., Thormählen I., Daloso D.M., Fernie A.R. (2017). The unprecedented versatility of the plant thioredoxin system. Trends Plant Sci..

[33] Okegawa Y., Motohashi K. (2015). Chloroplastic thioredoxin *m* functions as a major regulator of Calvin cycle enzymes during photosynthesis in vivo. Plant J..

[34] Nikkanen L., Rintamäki E. (2014). Thioredoxin-dependent regulatory networks in chloroplasts under fluctuating light conditions. Philos. Trans. R. Soc. Lond. B Biol. Sci..

[35] Yoshida K., Hara S., Hisabori T. (2015). Thioredoxin selectivity for thiol-based redox regulation of target proteins in chloroplasts. J. Biol. Chem..

[36] Mallén-Ponce M.J., Huertas M.J., Sánchez-Riego A.M., Florencio F.J. (2021). Depletion of *m*-type thioredoxin impairs photosynthesis, carbon fixation, and oxidative stress in cyanobacteria. Plant Physiol..

[37] Serrato A.J., Pérez-Ruiz J.M., Spínola M.C., Cejudo F.J. (2004). A novel NADPH thioredoxin reductase, localized in the chloroplast, which deficiency causes hypersensitivity to abiotic stress in *Arabidopsis thaliana*. J. Biol. Chem..

[38] Pérez-Ruiz J.M., Spínola M.C., Kirchsteiger K., Moreno J., Sahrawy M., Cejudo F.J. (2006). Rice NTRC is a high-efficiency redox system for chloroplast protection against oxidative damage. Plant Cell.

[39] Pérez-Ruiz J.M., Naranjo B., Ojeda V., Guinea M., Cejudo F.J. (2017). NTRC-dependent redox balance of 2-Cys peroxiredoxins is needed for optimal function of the photosynthetic apparatus. Proc. Natl. Acad. Sci. USA.

[40] González M., Delgado-Requerey V., Ferrández J., Serna A., Cejudo F.J. (2019). Insights into the function of NADPH thioredoxin reductase C (NTRC) based on identification of NTRC-interacting proteins in vivo. J. Exp. Bot..

[41] Gurrieri L., Fermani S., Zaffagnini M., Sparla F., Trost P. (2021). Calvin-Benson cycle regulation is getting complex. Trends Plant Sci..

[42] Lennartz K., Plücken H., Seidler A., Westhoff P., Bechtold N., Meierhoff K. (2001). HCF164 encodes a thioredoxin-like protein involved in the biogenesis of the cytochrome *b*_(6)_*f* complex in *Arabidopsis*. Plant Cell.

[43] Page M.L., Hamel P.P., Gabilly S.T., Zegzouti H., Perea J.V., Alonso J.M., Ecker J.R., Theg S.M., Christensen S.K., Merchant S. (2004). A homolog of prokaryotic thiol disulfide transporter CcdA is required for the assembly of the cytochrome *b*_6_*f* complex in *Arabidopsis* chloroplasts. J. Biol. Chem..

[44] Motohashi K., Hisabori T. (2006). HCF164 receives reducing equivalents froms stromal thioredoxin across the thylakoid membrane and mediates reduction of target proteins in the thylakoid lumen. J. Biol. Chem..

[45] Armbruster U., Correa Galvis V., Kunz H.H., Strand D.D. (2017). The regulation of the chloroplast proton motive force plays a key role for photosynthesis in fluctuating light. Curr. Opin. Plant Biol..

[46] Yamori W., Shikanai T. (2016). Physiological functions of cyclic electron transport around photosystem I in sustaining photosynthesis and plant growth. Annu. Rev. Plant Biol..

[47] Carrillo L.R., Froehlich J.E., Cruz J.A., Savage L.J., Kramer D.M. (2016). Multi-level regulation of the chloroplast ATP synthase: The chloroplast NADPH thioredoxin reductase C (NTRC) is required for redox modulation specifically under low irradiance. Plant J..

[48] Sekiguchi T., Yoshida K., Okegawa Y., Motohashi K., Wakabayashi K.I., Hisabori T. (2020). Chloroplast ATP synthase is reduced by both *f*-type and *m*-type thioredoxins. Biochim. Biophys. Acta Bioenerg..

[49] Uflewski M., Mielke S., Galvis V.C., von Bismarck T., Chen X., Tietz E., Ruß J., Luzarowski M., Sokolowska E., Skirycz A. (2021). Functional characterization of proton antiport regulation in the thylakoid membrane. Plant Physiol..

[50] Wang C., Yamamoto H., Narumiya F., Munekage Y.N., Finazzi G., Szabo I., Shikanai T. (2017). Fine-tuned regulation of the K^+^/H^+^ antiporter KEA3 is required to optimize photosynthesis during induction. Plant J..

[51] Shikanai T. (2016). Chloroplast NDH: A different enzyme with a structure similar to that of respiratory NADH dehydrogenase. Biochim Biophys. Acta.

[52] Peng L., Shikanai T. (2011). Supercomplex formation with photosystem I is required for the stabilization of the chloroplast NADH dehydrogenase-like complex in *Arabidopsis*. Plant Physiol..

[53] McKenzie S.D., Ibrahim I.M., Aryal U.K., Puthiyaveetil S. (2020). Stoichiometry of protein complexes in plant photosynthetic membranes. Biochim. Biophys. Acta Bioenerg..

[54] Nikkanen L., Toivola J., Trotta A., Guinea Diaz M., Tikkanen M., Aro E.M., Rintamäki E. (2018). Regulation of cyclic electron flow by chloroplast NADPH-dependent thioredoxin system. Plant Direct.

[55] Courteille A., Vesa S., Sanz-Barrio R., Cazalé A.C., Becuwe-Linka N., Farran I., Havaux M., Rey P., Rumeau D. (2013). Thioredoxin *m4* controls photosynthetic alternative electron pathways in *Arabidopsis*. Plant Physiol..

[56] Casano L.M., Martín M., Sabater B. (2001). Hydrogen peroxide mediates the induction of chloroplastic Ndh complex under photooxidative stress in barley. Plant Physiol..

[57] Queval G., Foyer C.H. (2012). Redox regulation of photosynthetic gene expression. Phil. Trans. R Soc. B.

[58] Lascano H.R., Casano L.M., Martín M., Sabater B. (2003). The activity of the chloroplastic Ndh complex is regulated by phosphorylation of the NDH-F subunit. Plant Physiol..

[59] Lin C.S., Chen J., Chiu C.C., Hsiao H., Yang C.J., Jin X.H., Leebens-Mack J., de Pamphilis C.W., Huang Y.T., Yang L.H. (2017). Concomitant loss of NDH complex-related genes within chloroplast and nuclear genomes in some orchids. Plant J..

[60] Nashilevitz S., Melamed-Bessudo C., Izkovich Y., Rogachev I., Osorio S., Itkin M., Adato A., Pankratov I., Hirschberg J., Fernie A.R. (2010). An orange ripening mutant links plastid NAD(P)H dehydrogenase complex activity to central and specialized metabolism during tomato fruit maturation. Plant Cell.

[61] DalCorso G., Pesaresi P., Masiero S., Aseeva E., Schünemann D., Finazzi G., Joliot P., Barbato R., Leister D. (2008). A complex containing PGRL1 and PGR5 is involved in the switch between linear and cyclic electron flow in *Arabidopsis*. Cell.

[62] Rantala M., Rantala S., Aro E.-M. (2020). Composition, phosphorylation and dynamic organization of photosynthetic protein complexes in plant thylakoid membrane. Photoch. Photobiol. Sci..

[63] Okegawa Y., Motohashi K. (2020). M-Type Thioredoxins regulate the PGR5/PGRL1-dependent pathway by forming a disulfide-linked complex with PGRL1. Plant Cell.

[64] Hertle A.P., Blunder T., Wunder T., Pesaresi P., Pribil M., Armbruster U., Leister D.L. (2013). PGRL1 is the elusive ferredoxin-plastoquinone reductase in photosynthetic cyclic electron flow. Mol. Cell.

[65] Okegawa Y., Tsuda N., Sakamoto W., Motohashi K. (2021). Maintaining the chloroplast redox balance through the PGR5-dependent pathway and the Trx system is required for light-dependent activation of photosynthetic reactions. Plant Cell Physiol..

[66] Dann M., Leister D. (2019). Evidence that cyanobacterial Sll1217 functions analogously to PGRL1 in enhancing PGR5-dependent cyclic electron flow. Nat. Commun..

[67] Deák Z., Sass L., Kiss É., Vass I. (2014). Characterization of wave phenomena in the relaxation of flash-induced chlorophyll fluorescence yield in cyanobacteria. Biochim. Biophys. Acta.

[68] Peltier G., Aro E.-M., Shinakai T. (2016). NDH-1 and NDH-2 plastoquinone reductase in oxygenic photosynthesis. Annu. Rev. Plant Biol..

[69] Tan Y., Zhang Q.S., Zhao W., Liu Z., Ma M.Y., Zhong M.Y., Wang M.X., Xu B. (2020). The highly efficient NDH-dependent photosystem I cyclic electron flow pathway in the marine angiosperm Zostera marina. Photosynth. Res..

[70] Hanke G., Mulo P. (2013). Plant type ferredoxins and ferredoxin-dependent metabolism. Plant Cell Environ..

[71] Voss I., Goss T., Murozuka E., Altmann B., McLean K.J., Rigby S.E., Munro A.W., Scheibe R., Hase T., Hanke G.T. (2011). FdC1, a novel ferredoxin protein capable of alternative electron partitioning, increases in conditions of acceptor limitation at photosystem I. J. Biol. Chem..

[72] Guan X., Chen S., Voon C.P., Wong K.B., Tikkanen M., Lim B.L. (2018). FdC1 and leaf-type ferredoxins channel electrons from Photosystem I to different downstream electron acceptors. Front. Plant Sci..

[73] Hanke G.T., Kimata-Ariga Y., Taniguchi I., Hase T. (2004). A post genomic characterization of *Arabidopsis* ferredoxins. Plant Physiol..

[74] Yamamoto H., Kato H., Shinzaki Y., Horiguchi S., Shikanai T., Hase T., Endo T., Nishioka M., Makino A., Tomizawa K. (2006). Ferredoxin limits cyclic electron flow around PSI (CEF-PSI) in higher plants--stimulation of CEF-PSI enhances non-photochemical quenching of Chl fluorescence in transplastomic tobacco. Plant Cell Physiol..

[75] Hanke G.T., Hase T. (2008). Variable photosynthetic roles of two leaf-type ferredoxins in *Arabidopsis*, as revealed by RNA interference. Photochem. Photobiol..

[76] Lehtimäki N., Lintala M., Allahverdiyeva Y., Aro E.M., Mulo P. (2010). Drought stress-induced upregulation of components involved in ferredoxin-dependent cyclic electron transfer. J. Plant Physiol..

[77] Blanco N.E., Ceccoli R.D., Vía M.V., Voss I., Segretin M.E., Bravo-Almonacid F.F., Melzer M., Hajirezaei M.R., Scheibe R., Hanke G.T. (2013). Expression of the minor isoform pea ferredoxin in tobacco alters photosynthetic electron partitioning and enhances cyclic electron flow. Plant Physiol..

[78] Zhao J., Qiu Z., Ruan B., Kang S., He L., Zhang S., Dong G., Hu J., Zeng D., Zhang G. (2015). Functional inactivation of putative photosynthetic electron acceptor ferredoxin C2 (FdC2) induces delayed heading date and decreased photosynthetic rate in rice. PLoS ONE.

[79] Sétif P., Fischer N., Lagoutte B., Bottin H., Rochaix J.D. (2002). The ferredoxin docking site of photosystem I. Biochim. Biophys. Acta.

[80] Müller P., Li X.P., Niyogi K.K. (2001). Non-photochemical quenching. A response to excess light energy. Plant Physiol..

[81] Pinnola A., Bassi R. (2018). Molecular mechanisms involved in plant photoprotection. Biochem. Soc. Trans..

[82] Malnoë A., Schultink A., Shahrasbi S., Rumeau D., Havaux M., Niyogi K.K. (2018). The plastid lipocalin LCNP is required for sustained photoprotective energy dissipation in *Arabidopsis*. Plant Cell.

[83] Nakano H., Yamamoto H., Shikanai T. (2019). Contribution of NDH-dependent cyclic electron transport around photosystem I to the generation of proton motive force in the weak mutant allele of pgr5. Biochim. Biophys. Acta Bioenerg..

[84] Hallin E.I., Guo K., Åkerlund H.E. (2015). Violaxanthin de-epoxidase disulphides and their role in activity and thermal stability. Photosynth. Res..

[85] Simionato D., Basso S., Zaffagnini M., Lana T., Marzotto F., Trost P., Morosinotto T. (2015). Protein redox regulation in the thylakoid lumen: The importance of disulfide bonds for violaxanthin de-epoxidase. FEBS Lett..

[86] Lu Y., Du J.-J., Yu Z.-B., Peng J.-J., Xu J.-N., Wang X.-Y. (2015). Identification of potential targets for thylakoid oxidoreductase *At*VKOR/LTO1 in chloroplasts. Protein Pept. Lett..

[87] Da Q., Sun T., Wang M., Jin J., Li M., Feng D., Wang J., Wang H.-B., Liu B. (2018). M-type thioredoxins are involved in the xanthophyll cycle and proton motive force to alter NPQ under low-light conditions in *Arabidopsis*. Plant Cell Rep..

[88] Naranjo B., Mignée C., Krieger-Liszkay A., Hornero-Méndez D., Gallardo-Guerrero L., Cejudo F.J., Lindahl M. (2016). The chloroplast NADPH thioredoxin reductase C, NTRC, controls non-photochemical quenching of light energy and photosynthetic electron transport in *Arabidopsis*. Plant Cell Environ..

[89] Brooks M.D., Sylak-Glassman E.J., Fleming G.R., Niyogi K.K. (2013). A thioredoxin-like/β-propeller protein maintains the efficiency of light harvesting in *Arabidopsis*. Proc. Natl. Acad. Sci. USA.

[90] Bru P., Nanda S., Malnoë A. (2020). A genetic screen to identify new molecular players involved in photoprotection qH in *Arabidopsis thaliana*. Plants.

[91] Nikkanen L., Guinea Díaz M., Toivola J., Tiwari A., Rintamäki E. (2019). Multilevel regulation of non-photochemical quenching and state transitions by chloroplast NADPH-dependent thioredoxin reductase. Physiol. Plant.

[92] Amstutz C.L., Fristedt R., Schultink A., Merchant S.S., Niyogi K.K., Malnoë A. (2020). An atypical short chain dehydrogenase/reductase functions in the relaxation of sustained energy dissipation in the antenna of photosystem II in *Arabidopsis*. Nat. Plants.

[93] Buchanan B.B., Balmer Y. (2005). Redox regulation: A broadening horizon. Annu. Rev. Plant Biol..

[94] Finazzi G., Barbagallo R.P., Bergo E., Barbato R., Forti G. (2001). Photoinhibition of *Chlamydomonas reinhardtii* in State 1 and State 2: Damages to the photosynthetic apparatus under linear and cyclic electron flow. J. Biol. Chem..

[95] Lemeille S., Willig A., Depége-Fargeix N., Delessert C., Bassi R., Rochaix J.-D. (2009). Analysis of the chloroplast protein kinase Stt7 during state transitions. PLoS Biol..

[96] Gabilly S.T., Dreyfuss B.W., Karamoko M., Corvest V., Kropat J., Page M.D., Merchant S.S., Hamel P.P. (2010). CCS5, a thioredoxin-like protein involved in the assembly of plastid *c*-type cytochromes. J. Biol. Chem..

[97] Shapiguzov A., Chai X., Fucile G., Longoni P., Zhang L., Rochaix J.D. (2016). Activation of the Stt7/STN7 kinase through dynamic interactions with the cytochrome *b_6_ f* complex. Plant Physiol..

[98] Wunder T., Liu Q., Aseeva E., Bonardi V., Leister D., Pribil M. (2013). Control of *STN7* transcript abundance and transient STN7 dimerisation are involved in the regulation of STN7 activity. Planta.

[99] Rintamäki E., Martinsuo P., Pursiheimo S., Aro E.-M. (2000). Cooperative regulation of light-harvesting complex II phosphorylation via the plastoquinol and ferredoxin-thioredoxin system in chloroplasts. Proc. Natl. Acad. Sci. USA.

[100] Thormälen I., Zupok A., Rescher J., Leger J., Weissenberger S., Groysman J., Orwat A., Chatel-Innocenti G., Issakidis-Bourguet E., Armbruster U. (2017). Thioredoxins play a crucial role in dynamic acclimation of photosynthesis in fluctuating light. Mol. Plant.

[101] Ancín M., Fernández-San Millán A., Larraya L., Morales F., Veramendi J., Aranjuelo I., Farran I. (2019). Overexpression of thioredoxin *m* in tobacco chloroplasts inhibits the protein kinase STN7 and alters photosynthetic performance. J. Exp. Bot..

[102] Pesaresi P., Hertle A., Pribil M., Kleine T., Wagner R., Strissel H., Ihnatowicz A., Bonardi V., Scharfenber M., Schneider A. (2009). *Arabidopsis* STN7 kinase provides a link between short- and long-term photosynthetic acclimation. Plant Cell.

[103] Pesaresi P., Pribil M., Wunder T., Leister D. (2011). Dynamics of reversible protein phosphorylation in thylakoids of flowering plants: The roles of STN7, STN8 and TAP38. Biochim. Biophys. Acta.

[104] Allen J.F., Santabarbara S., Allen C.A., Puthiyaveetil S. (2011). Discrete redox signalling pathways regulate photosynthetic light-harvesting and chloroplast gene transcription. PLoS ONE.

[105] Bandyopadhyay S., Gama F., Molina-Navarro M.M., Gualberto J.M., Claxton R., Naik S.G., Huynh B.H., Herrero E., Jacquot J.P., Johnson M.K. (2008). Chloroplast monothiol glutaredoxins as scaffold proteins for the assembly and delivery of [2Fe–2S] clusters. EMBO J..

[106] Talib E.A., Outten C.E. (2021). Iron-sulfur cluster biogenesis, trafficking, and signalling: Roles for CGFS glutaredoxins and BolA proteins. Biochim. Biophys. Acta Mol. Cell Res..

[107] Järvi S., Suorsa M., Aro E.-M. (2015). Photosystem II repair in plant chloroplasts-regulation, assisting proteins and shared components with photosystem II biogenesis. Biochim. Biophys. Acta.

[108] Lima-Melo Y., Gollan P.J., Tikkanen M., Silveira J.A., Aro E.-M. (2019). Consequences of photosystem-I damage and repair on photosynthesis and carbon use in *Arabidopsis thaliana*. Plant J..

[109] Allahverdiyeva Y., Suorsa M., Rossi F., Pavesi A., Kater M.M., Antonacci A., Tadini L., Pribil M., Schneider A., Wanner G. (2013). *Arabidopsis* plants lacking PsbQ and PsbR subunits of the oxygen-evolving complex show altered PSII super-complex organization and short-term adaptive mechanisms. Plant J..

[110] Sun X., Fu T., Chen N., Guo J., Ma J., Zou M., Lu C., Zhang L. (2010). The stromal chloroplast Deg7 protease participates in the repair of photosystem II after photoinhibition in *Arabidopsis*. Plant Physiol..

[111] Kley J., Schmidt B., Boyanov B., Stolt-Bergner P.C., Kirk R., Ehrmann M., Knopf R.R., Naveh L., Adam Z., Clausen T. (2011). Structural adaptation of the plant protease Deg1 to repair photosystem II during light exposure. Nat. Struct. Mol. Biol..

[112] Kato Y., Ozawa S.-I., Takahashi Y., Sakamoto W. (2015). D1 fragmentation in photosystem II repair caused by photo-damage of a two-step model. Photosynth. Res..

[113] Aro E.M., Suorsa A., Rokka A., Allahverdiyeva Y., Paakkarinen V., Saleem A., Battchikova N., Rintamäki E. (2005). Dynamics of photosystem II: A proteomic approach to thylakoid protein complexes. J. Exp. Bot..

[114] Lindahl M., Tabak S., Cseke L., Pichersky E., Andersson B., Adam Z. (1996). Identification, characterization and cloning of a homologue of the bacterial FtsH protease in chloroplasts of higher plants. J. Biol. Chem..

[115] Zaltsman A., Ori N., Adam Z. (2005). Two types of FtsH protease subunits are required for chloroplast biogenesis and photosystem II repair in *Arabidopis*. Plant Cell.

[116] Kato Y., Sakamoto W. (2018). FtsH protease in the thylakoid membrane: Physiological functions and the regulation of protease activity. Front. Plant Sci..

[117] Nishimura K., Kato Y., Sakamoto W. (2016). Chloroplast proteases: Updates on proteolysis within and across suborganellar compartments. Plant Physiol..

[118] Lindahl M., Kieselbach T. (2009). Disulphide proteomes and interactions with thioredoxin on the track towards understanding redox regulation in chloroplasts and cyanobacteria. J. Proteom..

[119] Hall M., Mata-Cabana A., Akerlund H.E., Florencio F.J., Schröder W.P., Lindahl M., Kieselbach T. (2010). Thioredoxin targets of the plant chloroplast lumen and their implications for plastid function. Proteomics.

[120] Karamoko M., Cline S., Redding K., Ruiz N., Hamel P.P. (2011). Lumen thiol oxidoreductase 1, a disulphide bond-forming catalyst, is required for the assembly of photosystem II in *Arabidopsis*. Plant Cell.

[121] Ferreira K.N., Iverson T.M., Maghlaoui K., Barber S., Iwata S. (2004). Arquitecture of the photosynthetic oxygen-evolving center. Science.

[122] Roberts I.N., Lam X.T., Miranda H., Kieselbach T., Funk C. (2012). Degradation of PsbO by the Deg protease HhoA is thioredoxin dependent. PLoS ONE.

[123] Lee K., Lee J., Kim Y., Bae D., Kang K.Y., Yoon S.C., Lim D. (2004). Defining the plant disulfide proteome. Electrophoresis.

[124] Marchand C., Le Marechal P., Meyer Y., Decottignies P. (2006). Comparative proteomic approaches for the isolation of proteins interacting with thioredoxin. Proteomics.

[125] Wyman A.J., Yocum C.F. (2005). Structure and activity of the Photosystem II manganese-stabilizing protein: Role of the conserved disulfide bond. Photosynth. Res..

[126] Nikitina J., Shutova T., Melnik B., Chernyshov S., Marchenkov V., Semisotnov G., Klimov V., Samuelsson G. (2008). Importance of a single disulfide bond for the PsbO protein of photosystem II: Protein structure stability and soluble overexpression in *Escherichia coli*. Photosynth. Res..

[127] Butenko Y., Lin A., Naveh L., Kupervaser M., Levin Y., Reich Z., Adam Z. (2018). Differential roles of the thylakoid luminal Deg protease homologs in chloroplast proteostasis. Plant Physiol..

[128] Havaux M. (2020). Plastoquinone in and beyond photosynthesis. Trends Plant Sci..

[129] Ksas B., Légeret B., Ferretti U., Chevalier A., Pospíšil P., Alric J., Havaux M. (2018). The plastoquinone pool outside the thylakoid membrane serves in plant photoprotection as a reservoir of singlet oxygen scavengers. Plant Cell Environ..

[130] Ksas B., Becuwe N., Chevalier A., Havaux M. (2015). Plant tolerance to excess light energy and photooxidative damage relies on plastoquinone biosynthesis. Sci. Rep..

[131] Carol P., Stevenson D., Bisanz C., Breitenbach J., Sandmann G., Mache R., Coupland G., Kuntz M. (1999). Mutations in the *Arabidopsis* gene IMMUTANS cause a variegated phenotype by inactivating a chloroplast terminal oxidase associated with phytoene desaturation. Plant Cell.

[132] Wu D., Wright D.A., Wetzel C., Voytas D.F., Rodermel S. (1999). The IMMUTANS variegation locus of *Arabidopsis* defines a mitochondrial alternative oxidase homolog that functions during early chloroplast biogenesis. Plant Cell.

[133] Kambakam S., Bhattacharjee U., Petrich J., Rodermel S. (2016). PTOX mediates novel pathways of electron transport in etioplasts of *Arabidopsis*. Mol. Plant.

[134] Cazzonelli C.I., Pogson B.J. (2010). Source to sink: Regulation of carotenoid biosynthesis in plants. Trends Plant Sci..

[135] Wang D., Fu A. (2016). The plastid terminal oxidase is a key factor balancing the redox state of thylakoid membrane. Enzymes.

[136] Thiers K.L.L., da Silva J.H.M., Sartori G.R., Dos Santos C.P., Saraiva K., Roque A., Arnholdt-Schmitt B., Costa J.H. (2019). Polymorphisms in plastoquinol oxidase (PTOX) from *Arabidopsis* accessions indicate SNP-induced structural variants associated with altitude and rainfall. J. Bioenerg. Biomembr..

[137] Feilke K., Ajlani G., Krieger-Liszkay A. (2017). Overexpression of plastid terminal oxidase in *Synechocystis* sp. PCC 6803 alters cellular redox state. Philos. Trans. R. Soc. Lond. B Biol. Sci..

[138] Borisova-Mubarakshina M.M., Vetoshkina D.V., Ivanov B.N. (2019). Antioxidant and signalling functions of the plastoquinone pool in higher plants. Physiol. Plant.

